# Nurses’ Knowledge about Delirium in the Group of Intensive Care Units Patients

**DOI:** 10.3390/ijerph19052758

**Published:** 2022-02-27

**Authors:** Sabina Krupa, Adriano Friganović, Ber Oomen, Snježana Benko, Wioletta Mędrzycka-Dąbrowska

**Affiliations:** 1 Institute of Health Sciences, College of Medical Sciences of the University of Rzeszow, Poland St. Warzywna 1A, 35-310 Rzeszow, Poland; sabinakrupa@o2.pl; 2 Department of Anesthesiology and Intensive Medicine, University Hospital Centre Zagreb, Kišpatićeva ul. 12, 10000 Zagreb, Croatia; 3 Department of Nursing, University of Applied Health Sciences, Mlinarska cesta 38, 10000 Zagreb, Croatia; adriano@hdmsarist.hr; 4 ESNO, European Specialist Nurses Organization, Pontanuslaan 12, 6821 HR Arnhem, The Netherlands; secretariat@esno.org; 5 Special Hospital for Lung Disease, Rockefellerova 3, 10000 Zagreb, Croatia; snjezanabenko@windowslive.com; 6 Faculty of Health Studies, International University of Rijeka, 51000 Rijek, Croatia; 7 Department of Anaesthesiology Nursing & Intensive Care, Faculty of Health Sciences, Medical University of Gdansk, Dębinki 7, 80-211 Gdańsk, Poland

**Keywords:** delirium, collective subject discourse, critical care

## Abstract

Background: Intensive Care Unit (ICU) delirium is a nonspecific, potentially preventable, and often reversible disorder of impaired cognition, which results from various causes in ICU patients. For appropriate management of delirium, early identification and risk factor assessment are key factors. Multidisciplinary collaboration and standardized care can enhance the recognition of delirium. Design: In this study, authors used the exploratory and descriptive study method. Method: The study was conducted in a group of 45 nurses of the cardiac intensive care unit. The department has 16 intensive care stations and is intended for patients after cardiac surgery who require intensive care in the postoperative period. Results: During the analysis the interviews, five Collective Subject Discourses were distinguished: signs and symptoms, physical restraint, use of sedatives, environment, and lack of education. Conclusion: Nurses have no knowledge of the factors contributing to the development of delirium, are unable to communicate with such patients and, most of all, do not know the consequences of the actions taken.

## 1. Introduction

The Diagnostic and Statistical Manual of Mental Disorders Fourth Edition (DSM-IV), describes delirium as an acute disturbance of consciousness associated with an acute mental or physical illness [1]. Both during the stay in the Intensive Care Unit (ICU) and during delirium, there are symptoms such as sleep disorders, changes in cognitive functions, anxiety, fear, and irritability [2]. There is a possibility of patients developing sleep disorders, changes in cognitive functions, anxiety, fear, or irritability. There is not much information in the literature on educational support for nurses about delirium. Nobody talks to nurses about what they know and what they do not. It is important to realize the importance of this topic. The care for life-threatening patients at ICUs is performed by a whole team of specialists who are specialized in the care of patients diagnosed with delirium [3]. Nurses’ knowledge about delirium is necessary in order to quickly detect any signs or symptoms. Due to the large number of members of the therapeutic team, it is necessary to conduct trainings in the field of delirium in the entire ICU team [4]. Due to the increasingly frequently diagnosed acute disturbance of consciousness in critically ill patients, it is important to conduct research that will answer the question: whether the knowledge of nurses about this disorder is sufficient and/or whether it needs to be expanded at further stages of professional development.

## 2. Materials and Methods

### 2.1. Design

In this study, authors used the exploratory and descriptive study method. Exploratory research is one that aims to provide insights into and an understanding of the problem faced by the researcher. Descriptive research, on the other hand, aims to describe something, mainly functions and characteristics. Authors used these methods in the study.

### 2.2. Setting

Collective subject discourse (CSD) is a systematic method of organization and tabulation of qualitative data, anchored in the theory of social representations, combining methodological rigor and the search for the essence of thought expressed in a given social context [5].

### 2.3. Participants

The study was conducted in a group of 45 nurses of the cardiac intensive care unit. The department has 16 intensive care stations and is intended for patients after cardiac surgery who require intensive care in the postoperative period. In the study group of 45 nurses, 42 met the inclusion criteria and agreed to participate in the study. Data collection took place from 4 December 2020 to 10 February 2021. The project received a positive opinion of the Bioethics Committee of the University of Rzeszów (Poland).

In a qualitative study, it would be difficult to describe the responses of all 42 nurses participating in the study, so we selected responses and described the results.

The study group was selected on purpose—cardiac surgery intensive care units nurses. Samples that were selected using probability sampling methods are more representative of the target population.

### 2.4. Inclusion and Exclusion Criteria

These included work experience at the CSICU for a minimum of 12 months, and giving informed consent to participate in the study.

### 2.5. Data Analysis

The data were obtained through partially structured interviews, which were conducted in two rounds—the first round took place before and the second after the delirium training at the ICU. Individual interviews were conducted during nurses’ breaks. During the interviews, nurses were asked to comment on what they thought about the approach and management of patients who developed delirium. Nurses were not asked specific questions. The interviews were recorded, transcribed, and returned to the participating nurses for final approval. Nurses received their code, which was marked with the letter P (nurse), followed by a number that constituted the next number of the interview. The interview lasted from 23 to 30 min.

### 2.6. Training Plan

The training was conducted in a group of all CSICU nurses participating in the project, in a conference room that was remote from the CSICU where they work. The training consisted of several stages.

- In the first part of the training, nurses were introduced to the problem of delirium in Poland and around the world, as well as the factors influencing the formation of delirium and its complications.
- In the second stage, the nurses watched a short 18 min video recording of a conversation with a patient who had experienced delirium at the ICU.
- Another element of the training included listening to a recording that was recorded during one of the CSICU night shifts. On the recording, you can hear conversations, laughter, shuffling chairs, alarms of cameras, closing lockers, etc.
- Nurses, after watching the film and listening to the recording, shared their own experiences of working with delirium patients. Nurses were informed that their statements were confidential in the group they were in.
- The next stage was related to making nurses aware of the consequences of decisions made in caring for a delirious patient.
- The effects and side-effects of sedative and analgesic drugs were discussed.
- During the training, they were presented with the legal consequences of using drugs without a doctor’s order. They were also presented with various tools that can be used to assess delirium in the ICU (CAM-ICU, 4AT, NuDesc).
- Particular attention was paid to how to talk to delirious patients and their families. At the end of the meeting, nurses received an information booklet, produced in collaboration with Health Improvement Scotland. The brochure is available at https://ptpaio.pl/dokumenty/96.pdf [6]. As a result of the training, there was a discussion that the training brought a lot of information about delirium, actions, and their consequences, about which nurses had not known so far.

The methodological features of the Collective Subject Discourse (CSD) were passages or key elements (KE) that describe the essence of the content of specific discourses. Central Ideas (CI) briefly and succinctly described the meaning of the EC in each of the discourses. A coherent set of KE and CI were called categories. Anchoring (AC) was described when the nurse used a generic statement to describe a specific situation. Collective Subject Discourse (CSD) consisted of both KEs and CIs that belong to the same category [5].

From the researcher’s point of view, in order to isolate the EC from a specific discourse, the collected material (interview) was analyzed, and in the next stage, all the most important elements were identified. In the process of analysis, the commission was distinguished, and for it, the CI. In the next step, the CI elements that were consistent with each other were grouped. For each group, a CI was defined that was unique, and then the category was determined. The Collective Subject Discourse (CSD) consisted of KEs and CIs that belong to the same category.

## 3. Results

**Figure 1:**
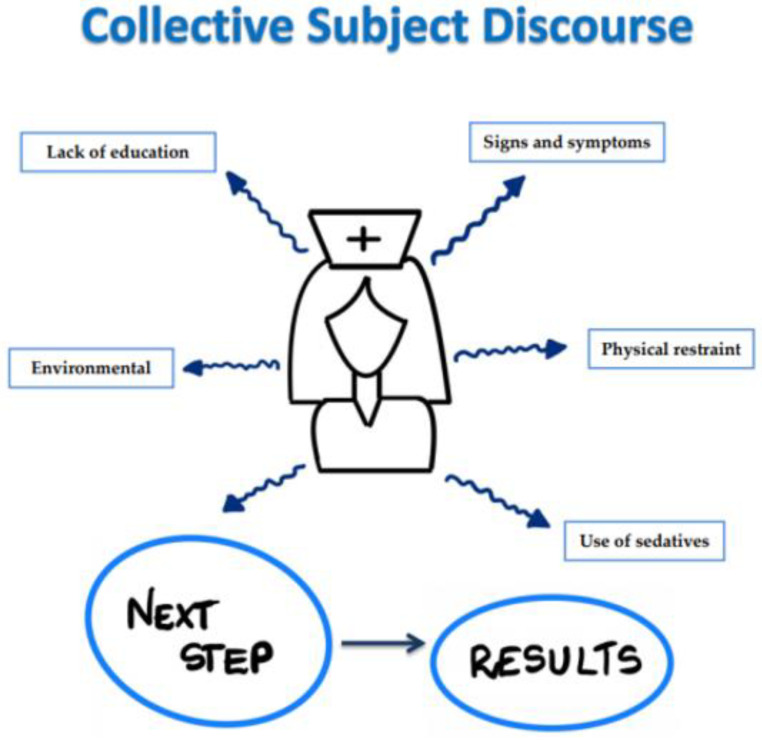
Collective Subject Discourses.

During the analysis of the interviews, five Collective Subject Discourses were distinguished (Figure 1).

The items included in individual dimensions were signs and symptoms, physical restraint, use of sedatives, and lack of education. In collective dimensions, we had an environment. Below are fragments from anonymous statements of the nurses’ conversations.

### 3.1. First Round

#### 3.1.1. CSD 1. Signs and Symptoms

Patients with delirium often have no contact with the real world. They are secluded. When I asked them to do something, they act as if they don’t understand it. Often these sick people experience anxiety, call for help, cry and shout at the same time. Patients cannot answer simple questions such as where they are and why. While on call, each of us is vigilant and observes the delirious patient.

#### 3.1.2. CSD 2. Physical Restraint

We cannot keep the patient safe unless we use physical force. Sometimes when I look at patients, I get the feeling that they are too embarrassed, but I know that when I trust them, they will hurt themselves and other ward employees. Especially during night shifts, the nurse must make the decision to use physical restraint herself before the doctor arrives. Sometimes a few people aren’t enough to keep the patient still. There are no specific guidelines on what a nurse can do if a patient develops delirium. Sometimes I release a patient, but only in consultation with the rest of the treatment team. Then I never approach the patient alone, because I am afraid of his reaction, I am not able to predict it. If the patient would hurt himself after being released, it would be my fault because he is under my constant care. It is best to use sedatives by continuous infusion. Then the patient is calm and I have the opportunity to work calmly. More than once, I prefer to give the patient sedative medications and use physical coercion than to hurt the patient. A calm and sedated patient, is a better patient. Any extubation carries a risk of delirium.

#### 3.1.3. CSD 3. Use of Sedatives

In Poland, there are no strict recommendations what drugs should be used in patients who develop delirium. Patients often feel more anxious after taking benzodiazepines. They require more attention. More than once, I have to give a bolus of propofol when the patient becomes delirious. Most often it is at night. Sometimes there is an overdose of sedative medications because I increase the dose when the doctor goes to sleep and wants to be calm. When a patient is very agitated, I have no patience with him. A sedated patient is an easier patient and does not interfere with my work.

#### 3.1.4. CSD 4. Environmental

Sometimes we are noisy in the room where we work with patients. I know this could contribute to the development of delirium in patients. Talking and laughing at night would throw anyone off balance. ICU patients are particularly susceptible to delirium when they suffer from noise, lit lights, and staff conversations. A patient in delirium often wakes other patients, which favors the onset of delirium in subsequent patients.

#### 3.1.5. CSD 5. Lack of Education

In my opinion, there are no specific guidelines for dealing with patients with delirium. We ourselves have to deal with situations where the patient is delirious. We cannot legally give him medication, we cannot use physical force, and yet we do it to keep the patient and ourselves safe. Most of us do not read the available publications on dealing with patients with delirium. There is no training on how to deal with delirious patients. In order to deal with patients with delirium, experience is necessary. When I am with the new nurse in the ward, I am afraid of being on duty, because I know that she has no experience, and no one has educated her on how to deal with patients with delirium. I often tell patients to be reassured that they are at the ICU, that we can see them all the time, we have the possibility of constant monitoring. But no one taught me that, I understood myself that sometimes it helps. There are no guidelines that say that a patient needs items that help him calm down—his favorite music, a prayer book or a photo of a loved one. Often, families do not know how to deal with a patient who has undergone a delirium. In addition to staff education, there is a great need to educate the family and the patients themselves about the possible occurrence of delirium.

### 3.2. Second Round

#### 3.2.1. CSD 1. Signs and Symptoms

The delirious patients are not easy to care. They are often unaware of their health and condition. They are agitated, screaming, have no awareness of where they are. You must always carefully observe such a patient. It is best to identify delirium at the very first stage of symptoms. Tools adapted to recognize delirium can serve this purpose.

#### 3.2.2. CSD 2. Physical Restraint

A delirious patient can be dangerous to both himself and the staff. Keep in mind that physical coercion is not always a good solution. It is the last resort in the fight against delirium. The consequences of using physical coercion are enormous, ranging from physical to legal. The application of physical coercion involves the initiation of a procedure that should be followed by every department. You should never make the decision to restrain yourself a patient. The decisions should be made by the doctor on duty in consultation with the entire team. Before applying physical force, try to talk to the patient and his family. Sometimes the presence of relatives allows patients to calm down, feel safe and thus limit the occurrence of full-blown delirium. However, if physical restraint is necessary, it should be carried out in accordance with the procedure. We are currently aware of having such a procedure.

#### 3.2.3. CSD 3. Use of Sedatives

It is true that there is no single scheme for conducting pharmacotherapy in a patient with delirium, each doctor conducts the therapy of the patient in accordance with his knowledge and belief. The doctor will provide information on the need for treatment. Medicines administered to patients must be recorded and dosing should be strictly in accordance with the doctor’s prescription. Do not increase the dose of opioids and other sedatives on your own. It may result in respiratory and hemodynamic failure. Self boluses of propofol are prohibited. If we suspect that the patient requires an increase or a change of medications, consult a physician. Thanks to my knowledge of medications and management, I have more patience if a person develops delirium. I treat patients without fear that I will not be able to cope with them.

#### 3.2.4. CSD 4. Environmental

Since the training, the medical staff has been quieter during the night shift. The patient can rest and relax. I have noticed that when it is quiet, patients do not require sedation, and delirium is much less common. Turning off the lights also became a routine during night shifts.

#### 3.2.5. CSD 5. Lack of Education

There are many tools to assess delirium that can be used already at the stage of patient admission to the ICU. Knowing how to conduct such an examination, makes me feel safer and more confident. I know what drugs to prescribe for different types of delirium. Sometimes I suggest to the doctor that an opioid is not indicated and that the supply of haloperidol is sufficient. Doctors agree and they can see that I am competent about the procedure. The video watched during the training showed me that patients remember tying them up and screaming. Now I know how to talk to patients. This was the first time I attended such a delirium training and it interested me very much. After the training, I became interested in the subject of delirium and I can say that it is a very difficult and underestimated topic. I believe that this type of training should be cyclical. The new scales that I learned are easy to use and do not pose a problems for me during the test.

## 4. Discussion

According to the Diagnostic and Statistical Manual of Mental Disorders, Fourth Edition, delirium is a disorder of consciousness that is characterized by cognitive alteration and limited attention to the environment. Elements such as memory, perception, and understanding of the environment are also shaken. Delirium is growing rapidly. Risk factors for delirium have been described in numerous studies. This group includes: old age, cognitive disorders, hearing and vision disorders, sleep disorders, immobilization, dehydration, and the use of sedatives [1]. The CSD1 study of rounds one and two showed that Polish CSICU nurses are able to identify delirium in patients. Basic delirium symptoms such as confusion, disorientation, and agitation are well recognized by nurses. On the other hand, Voyer, F.M. et al. described that the diagnosis of delirium in a patient is a significant problem for nurses. Worldwide strategies aim to improve delirium recognition. Thanks to this, there is a great chance to reduce the adverse effects of delirium [7]. The nurses in the first round, in the CSD2 study, mentioned the necessity to use physical restraint in the case of delirium. Physical restraint is needed to secure the patient safety, and as a last intervention and as short as possible. It is known that sometimes ‘physical restraining measures’ need to be professional, so some guidance and training on how to do this is also recommended. Despite the lack of guidelines that give the green light to make decisions about the implementation of physical compulsion, nurses make their own decisions on this topic. Salluch, J.I.F. et al. described that many centers undertake work on the assessment of delirium in the ICU, but nevertheless, there are no specific procedures implemented that can prevent or eliminate the risk of delirium [8]. In the present study, it was observed that the nurses had no concerns about the need to increase the dose of sedative drugs. This could be due to the lack of knowledge about the complications of such a procedure. None of the nurses in round one mentioned that they were worried about the consequences if a patient was immobilized. In the second round of the study, the nurses mentioned that they are now aware that immobilization of the patient may further worsen the patient’s condition in the event of delirium, and the use of higher doses of sedation without orders is against the law and has a destructive effect on the patient’s condition. The nurses in the second round emphasized that it is better to prevent signs of delirium at the very beginning than to use methods that are inconsistent with knowledge, law, and conscience. Ribeiro, R.S. et al. noted in his study the nurses were concerned about the inappropriate use of physical force. They were afraid of responsibility in the event of an adverse event [9]. The nurses in Round 1 and Round 2, in the CDS3 study, found that nurses had a large problem with delirium patients. The respondents stated that a patient requiring sedation is a patient who manages better because they do not cause problems. In the first round, none of the respondents pointed out that prolonged sedation negatively affects the length of stay in the ICU and affects the general parameters of the patient. In the first round, the nurses admitted that they often increase the doses of sedatives on their own, because otherwise, the patient and the staff are exposed to danger from an agitated patient. In round two, the nurses highlighted the fact that they are more aware of the side-effects of prolonged sedation after training. Krupa, S. et al. in his publication described various drug regimens used in patients with delirium. In his review, however, there is no single pattern used in the world marked. Each of the authors of the review listed different medications and drug regimens in the event of delirium [2]. Bourne, R.S. emphasized in his work that excessive sedation carries the risk of hemodynamic instability, prolongation of mechanical ventilation, and recurrence of delirium. Opioids are known for their side-effects after long-term use in ICUs [10]. In the author’s own study, none of the nurses mentioned these effects in the first round of the study, so it can be concluded that the nurses did not know about it or were not fully trained in the side-effects of the drugs used. Svenningsen described his study in Denmark as crucial to the sedatives used. He proved that in patients who were sedated with fentanyl, the risk of an increase in delirium was ten times higher [11]. Alexander, E. in his publication emphasized that the use of benzodiazepines may cause agitation, aggression, hostility, but also confusion [12].

The nurses in the first round in the CDS4 study admitted themselves that the resting environment they create for their patients is unfavorable. In the second round, in the same question, the nurses emphasized that after listening to the recording with sounds from their ward on night duty, they felt uncomfortable knowing that all these sounds and conversations were heard by a patient who was in a life-threatening condition. Nurses rated the element of environmental education as one of the most important and was not paid attention to before. Lewandowska, K. et al., in his study, emphasized that there is no intensive care unit in which the alarms are not audible. She emphasized that nurses perceive alarms as a very bothersome element that is ubiquitous in patient care at the ICU [13]. Ribeiro, R.S. et al. mentioned that high noise levels are associated with the large number of workers present at the ICU. According to his research, nursing consoles are often too close to patients’ beds, but there is no other way to react quickly in the event of deterioration of the patient’s health [9]. Macedo described that high noise levels are destructive not only for patients, but also for ICU colleagues. The main effects of excessive noise by the author include inter alia, irritability, muscle contractions, increased heart rate and blood pressure, and even deterioration of sleep quality [14]. Silva, C.L. et al. pointed out that thanks to normal conversations, switching on the lights, etc., the patients have at least some degree of maintenance of the circadian rhythm, which is often disturbed by their stay in the ICU. The use of corrective glasses and hearing aids may also prevent delirium in this group of patients [15]. One of the most frequently emphasized elements of the discussion in the study was the issue of education. The first round, which took place before the intra-ward training, showed that nurses reported the problem of a lack of education on the topic of delirium. Many actions taken by them are illegal, which is the result of a lack of knowledge about the consequences of such actions. Training staff in the diagnosis and treatment of delirium has a significant impact on understanding the consequences of a lack of knowledge in this field [16]. In his publication, Forsgren, L.M. et al. described a study in which the sensitivity of delirium diagnosis in the ICU without the use of screening tools is low [17]. Timely and correct delirium detection is closely related to the level of experience and education of the nursing team. Faria, R.S.B. et al. strongly emphasized that education is the basis for effective intervention. The same author emphasized in his work that there are proposals in the world to use preliminary algorithms that can prevent the occurrence of delirium [18]. These algorithms include training first. In subsequent stages, it is the identification of individual factors related to the patient’s weaknesses, minimization of conditions imposed by the hospital environment, severe diseases, interventions with the use of pharmacotherapy, as well as daily monitoring of sedation and delirium [19,20]. 

## 5. Study Limitations 

Limitations are related to the nurses’ lack of knowledge about delirium. Additionally, there is no regular training on delirium. Nurses do not have sufficient educational materials and are not well prepared to work with patients with delirium. The application of physical restraint to patients in ICU encompasses ethical, legal, physical, and psychological considerations. However, there is no mandatory training of personnel in this field.

## 6. Conclusions

Nurses do not have enough knowledge and competencies of the factors contributing to the development of delirium, are unable to communicate with such patients and, most of all, do not know the consequences of the actions taken. After watching the video and listening to the video, the nurses were surprised and somewhat embarrassed by the video, but admitted that they never paid attention to the need for patients to recover during this time. One may be tempted to conclude that the nurses, due to the lack of education, do not feel comfortable when caring for a delirious patient. In comprehensive training, it is important to present methods of delirium prevention, including proper communication with the patient and his family.

## 7. Implications for Practice

During the training conducted in the department, it appears that there are no implemented tools to assess delirium. There are currently no effective delirium tracking tools in place, but there is also no training that can increase staff awareness of delirium detection. Interventions related to the education of nurses can significantly contribute to the improvement of the ability to deal with a difficult patient, which is definitely a delirious patient. In addition to delirium education, training in pain assessment and sedation is essential.

## Data Availability

A dataset will be made available upon request to the corresponding authors one year after the publication of this study. The request must include a statistical analysis plan.
